# Can We Use Urinary Cytokine/Chemokine Analysis in Discriminating Ulcer-Type Interstitial Cystitis/Bladder Pain Syndrome?

**DOI:** 10.3390/diagnostics12051093

**Published:** 2022-04-27

**Authors:** Yuan-Hong Jiang, Jia-Fong Jhang, Hann-Chorng Kuo

**Affiliations:** Department of Urology, Hualien Tzu Chi Hospital, Buddhist Tzu Chi University, Buddhist Tzu Chi Medical Foundation, Hualien 43000, Taiwan; redeemerhd@gmail.com (Y.-H.J.); alur1984@hotmail.com (J.-F.J.)

**Keywords:** interstitial cystitis, painful bladder syndrome, inflammation, cytokines, oxidative stress biomarkers

## Abstract

Purpose: Interstitial cystitis/bladder pain syndrome (IC/BPS) has ulcer (HIC) and non-ulcer subtypes. Differentiation of these two subtypes could only be based by cystoscopy. This study analyzed the urinary cytokines and chemokines among IC/BPS subtypes and controls for discriminating HIC from non-HIC and controls. Materials and Methods: A total of 309 consecutive patients with clinically diagnosed IC/BPS were enrolled. All patients received cystoscopic hydrodistention under anesthesia and urine samples were collected prior to the procedure. Enrolled patients were classified into subtypes based on the glomerulation grade, maximal bladder capacity (MBC), and presence of Hunner’s lesion. Inflammation-related cytokines and chemokines in urine samples, including interleukin-8 (IL-8), C-X-C motif chemokine ligand 10 (CXCL10), monocyte chemoattractant protein-1 (MCP-1), brain-derived neurotrophic factor (BDNF), eotaxin-1 (eotaxin), IL-6, macrophage inflammatory protein-1 beta (MIP-1β), regulated upon activation, normally T-expressed, and presumably secreted (RANTES), tumor necrosis factor-alpha (TNF-α), and prostaglandin E2 (PGE2) were assayed using commercially available microspheres with the Milliplex^®^ Human Cytokine/Chemokine Magnetic Bead-based Panel kit. The clinical data and urine levels of analytes between IC/BPS patients and controls, and among HIC, non-HIC, and controls were analyzed. Results: Among the 10 proteins, MCP-1, eotaxin, MIP-1β, TNF-α, and PGE2 were significantly different between IC/BPS and control, while IL-8, CXCL10, BDNF, IL-6, and RANTES were significantly higher in HIC than non-HIC patients. The receiver operating characteristic curve was used to analyze each urine biomarker in the patients with IC/BPS and controls. Among the 10 urine biomarkers, MIP-1β and TNF-α had an area under curve of >0.70 to predict IC/BPS from controls, however, the predictive values of these urine biomarkers to predict HIC from non-HIC were low. Combined cut-off values of MIP-1β and TNF-α can only have a 50% sensitivity and 39.6% specificity in identifying HIC from non-HIC. Conclusion: The results of this study demonstrate that urine cytokines and chemokines may be useful to discriminate patients with HIC from controls. An elevation of urine levels of IL-8, CXCL 10, BDNF, IL-6, and RANTES in IC/BPS patients should prompt physicians to consider the diagnosis of HIC.

## 1. Introduction

Interstitial cystitis/bladder pain syndrome (IC/BPS) is characterized by urinary urgency and frequency, usually accompanied by pelvic pain and nocturia, in the absence of bacterial infection or identifiable pathology [1]. IC/BPS is associated with a decrease in work productivity, emotional change, sleep, and sexual dysfunction, which have negative impact on the quality of life [2]. There are different subtypes of IC, including the ulcer and non-ulcer types, which may have distinct histopathology and clinical characteristics [3,4]. Ulcer-type IC is characterized by a chronic eruptive Hunner’s lesion (HIC) in the bladder wall under cystoscopic inspection [4]. The Hunner’s lesion is formed by the underlying lymphoplasmic inflammation with severe urothelial erosion. Patients with HIC are characterized by small bladder capacity and severe bladder pain [5]. In an analysis using computed tomography, the bladder wall showed focal or diffused thickness in Hunner’s lesions [6], and the lesions are likely to be associated with Epstein–Barr virus (EBV) infection [7]. Among the IC/BPS patients without Hunner’s lesion (non-HIC), different grades of glomerulation and maximal bladder capacity (MBC) under cystoscopic hydrodistention have been linked to distinct bladder characteristics and treatment outcomes [8]. Under cystoscopic hydrodistention, some patients may have a reduced MBC, mild-to-severe glomerulations; however, some do not show these features. Patients with high MBC and a low glomerulation grade after hydrodistention had more medical comorbidities, but a significantly higher rate of satisfactory treatment outcome [8].

In patients with IC/PBS, researchers have found specific histopathologies, including abnormalities of the urothelium, changes in the nerve function within the bladder wall, and over-activation of mast cells [9,10]. These findings suggest that several possible pathogenetic developmental pathways are involved in IC/PBS, including chronic inflammation and urothelial dysfunction [11]. Histological studies using tissue biopsy samples obtained from patients with IC/PBS have consistently reported signs of inflammation as evident from degranulated mast cells, infiltration of mast cells, macrophages, and neutrophils [12]. The role of mast cells in the pathogenesis of IC-like bladder inflammation had been demonstrated in a transgenic autoimmune cystitis mice model, and the inflammation could be reversed by treating with a mast cell membrane stabilizer [13]. HIC is an inflammatory disorder characterized by pancystitis with B cell abnormalities and epithelial denudation, while non-HIC shows minimal histological changes [14]. Our recent study also revealed that the bladder histopathological findings were associated with clinical parameters and differences in patient-reported treatment outcomes [15]. IC/BPS patients without remarkable bladder histopathological findings had less favorable treatment outcomes compared with those who did. Previous study using symptom-based clustering also found some IC/BPS patients have severe psychosocial comorbidities and non-pelvic pain [16]. Searching for tissue biomarkers to differentiate IC/BPS subtypes may help in treatment decisions.

Previous research studies on urine or serum biomarkers did not yield significant findings in discriminating different IC/BPS subtypes or clinical characteristics [17,18]. The levels of urinary biomarkers may not be elevated in all IC/PBS bladders, despite the presence of typical bladder pain symptoms and cystoscopic findings [12,19,20]. Recent studies revealed that mast cell density is not correlated with the duration of symptom amelioration after complete transurethral resection of HIC [21]. Moreover, overall urine markers were not associated with biopsy findings [22]. Therefore, we speculated that the histological findings, urinary biomarkers, and clinical presentations in IC/PBS may be heterogeneous. There is an utmost need to search and identify non-invasive markers for discriminating different subtypes of IC/BPS. Such markers would be useful for the early accurate diagnosis and effective treatment of patients with IC/BPS, particularly those with HIC.

Recent studies on urine biomarkers for the detection of IC/BPS revealed that the profiles of urine cytokines and chemokines differed significantly among the study and control groups [23]. Furthermore, previous studies reported that hypoxia occurred in IC/BPS bladders, and the expression of hypoxia-inducible factor-1 alpha (HIF1α) and vascular endothelial growth factor (VEGF) in the bladder was increased [24,25]. However, the results of different studies on urine biomarkers for the diagnosis of IC/BPS subtypes were inconsistent, possibly due to varied diagnostic criteria for patient enrollment, the condition of the bladder at the time of urine collection, and disease severity [18].

Cytokines and chemokines are redundant secreted proteins that regulate and determine the nature of immune responses and control immune cell trafficking and the cellular arrangement of immune organs [26]. Analysis of urine cytokines and chemokines may provide evidence of the immune response in bladder inflammation of IC/BPS. The aim of this study was to analyze the urinary cytokines and chemokines among different subtypes of IC/BPS and controls, and discriminate patients with HIC from non-HIC patients and controls.

## 2. Materials and Methods

### 2.1. Patients

From February 2014 to December 2021, we enrolled 309 consecutive patients with clinically diagnosed IC/BPS at the Department of Urology of Hualien Tzu Chi Hospital, Hualien, Taiwan. The diagnostic criteria for IC/BPS were based on the proposed guidelines of the European Society for the Study of Interstitial Cystitis (ESSIC) with the exclusion of similar diseases [27]. This study was approved by the Institutional Review Board (IRB) and Ethics Committee of Buddhist Tzu Chi General Hospital (IRB approval numbers: 105-25-B, 105-31-A, 107-175-A). All study and control patients were informed regarding the rationale and procedures of this study, and written informed consent was provided on the part of patients. Consent was waived for patients with urine samples collected in previous clinical trials.

**Figure 1:**
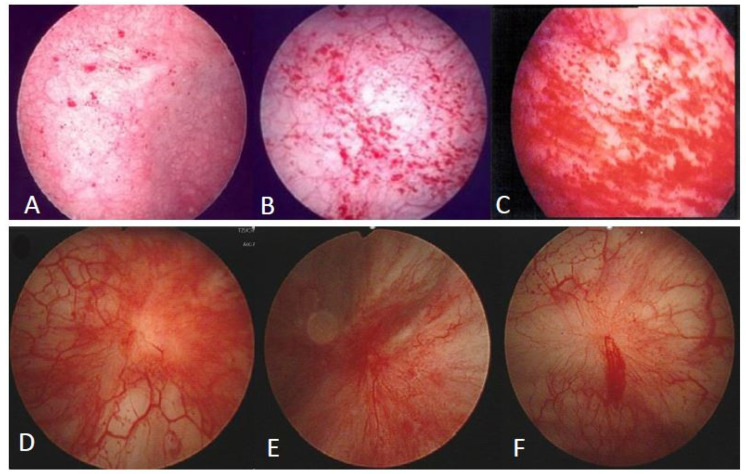
Findings of cystoscopic hydrodistention in patients with interstitial cystitis (IC) subtypes: (**A**) Non-Hunner’s IC (Non-HIC) with grade 0–1 glomerulation; (**B**) Non-HIC with grade 2 glomerulation; (**C**) Non-HIC with grade 3 glomerulation; (**D**–**F**) Hunner’s IC.

The patients were admitted for cystoscopic hydrodistention under general anesthesia. Hydrodistention was performed under an intravesical pressure of 80 cm H_2_O for 10 min, and the bladder was slowly evacuated. The bladder was carefully inspected for the formation of petechia, glomerulations, splotch hemorrhage, mucosal fissures, or presence of Hunner’s lesion [28]. The glomerulation grade was classified according to the appearance of glomerulations as follows: 0, none; 1, less than half of the bladder wall; 2, more than half of the bladder wall; or 3, severe waterfall bleeding. Patients with Hunner’s lesions with or without glomerulation were classified as having HIC (Figure 1). A total of 30 women with genuine stress urinary incontinence and without other storage or voiding dysfunction were included as controls. The inclusion and exclusion criteria were identical to those described in detail in our previous study [23].

In patients with IC/BPS, the assessment of clinical symptoms included the O’Leary–Saint symptom score, interstitial cystitis symptom index (ICSI), interstitial cystitis problem index (ICPI), and visual analog scale (VAS) pain score. The grade of glomerulation and MBC under anesthesia on cystoscopic hydrodistention were recorded. Patients with IC/BPS were classified into four clinical subtypes based on the grade of glomerulation and MBC: (1) patients with glomerulation grade ≤ 1 and MBC ≥ 760 mL; (2) patients with glomerulation grade ≤ 1 and MBC < 760 mL; (3) patients with glomerulation grade ≥ 2 and MBC ≥ 760 mL, and (4) patients with glomerulation grade ≥ 2 and MBC < 760 mL [8].

### 2.2. Urine Biomarker Investigation

Urine samples were collected from all study patients and controls prior to cystoscopic hydrodistention. Urine was self-voided when the patients had a full bladder sensation. Urinalysis was simultaneously performed to confirm an infection-free status before storage of the urine samples. Urine (50 mL) was immediately placed on ice and transferred to the laboratory for preparation. The samples were centrifuged at 1800 rpm for 10 min at 4 °C. The supernatant was separated into aliquots in 1.5 mL tubes (1 mL per tube) and preserved in a freezer at −80 °C. Before further analyses were performed, the frozen urine samples were centrifuged at 12,000 rpm for 15 min at 4 °C, and the supernatants were used for subsequent measurements. The procedures utilized for the analysis of urine cytokines and chemokines were previously described [29].

### 2.3. Cytokine and Chemokine Assay

Inflammation-related cytokines in urine samples were assayed using commercially available microspheres with the Milliplex^®^ Human Cytokine/Chemokine Magnetic Bead-based Panel kit (Millipore, Darmstadt, Germany). The selection of target analytes was based on previously reported significant urine cytokines and chemokines found in patients with IC/BPS versus controls. We selected 10 targets, namely interleukin-8 (IL-8), C-X-C motif chemokine ligand 10 (CXCL10), monocyte chemoattractant protein-1 (MCP-1), brain-derived neurotrophic factor (BDNF), eotaxin, IL-6, macrophage inflammatory protein-1 beta (MIP-1β), regulated upon activation, normally T-expressed, and presumably secreted (RANTES), tumor necrosis factor-alpha (TNF-α), and prostaglandin E2 (PGE2). Their levels were measured with the multiplex kit (catalog number HCYTMAG-60K-PX30). The following laboratory procedures for the quantification of these targeted analytes were performed according to the instructions provided by the manufacturer and were similar to those reported in our previous study [23,29].

### 2.4. Statistical Analysis

Continuous variables are represented as means ± standard deviations, and categorical data are represented as numbers and percentages. Cytokines with mean values below the minimum detectable concentrations as per the assay manufacturer were excluded for further analysis. Receiver operating characteristic (ROC) curves and cut-off values were generated for the ability of each targeted analyte to distinguish patients with IC/BPS from controls, as well as patients with HIC from all non-HIC patients; the area under the ROC curve (AUC) was calculated. The levels of urine biomarkers were compared between patients with IC/BPS and controls, among subtypes of non-HIC patients with different MBC, different glomerulation grade, and HIC, and among different IC/BPS subgroups and controls. The sensitivity, specificity, positive predictive value (PPV), and negative predictive value (NPV) of each urine biomarker for the diagnosis of IC/BPS and HIC were also calculated. All calculations were performed using SPSS Statistics for Windows, Version 20.0 (IBM Corp., Armonk, NY, USA). The *p*-values < 0.05 denoted statistically significant differences.

## 3. Results

A total of 309 patients with IC/BPS and 30 controls were enrolled in this study. The mean age of patients with IC/BPS and controls was 53.1 ± 13.4 and 57.7 ± 10.1 years, respectively (*p* = 0.068). Among patients with IC/BPS, 261 were women and 48 were men; all controls were women. At the time of urine collection and cystoscopic hydrodistenion, the mean ICSI was 11.1 ± 4.56, ICPI was 10.9 ± 3.82, VAS was 4.48 ± 2.87, and MBC was 721.8 ± 186.5 mL. Following cystoscopic hydrodistention, IC/BPS patients were divided into five subgroups with different MBC and glomerulations grades: (1) glomerulation ≤ 1, MBC > 760 mL (*n* = 85); (2) glomerulation ≤ 1, MBC ≤ 760 mL (*n* = 70); (3) glomerulation ≥ 2, MBC > 760 mL (*n* = 41); (4) glomerulation ≥ 2, MBC ≤ 760 mL (*n* = 89), and (5) HIC (*n* = 24).

**Figure 2:**
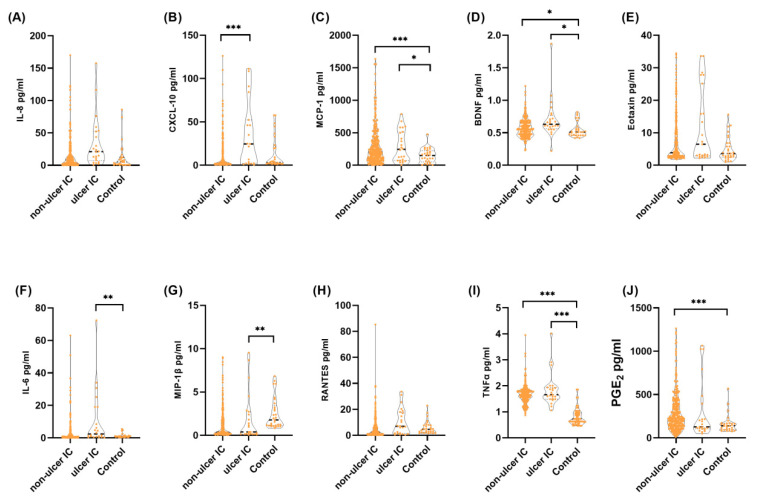
Violin plots of the levels of urine biomarkers among different subgroups of patients with IC/BPS and controls. Urine levels of (**A**) IL-8; (**B**) CXCL10; (**C**) MCP-1; (**D**) BDNF; (**E**) eotaxin; (**F**) IL-6; (**G**) MIP-1β; (**H**) RANTES; (**I**) TNF-α, and (**J**) PGE2. Among the biomarkers, MCP-1, eotaxin, MIP-1β, TNF-α, and PGE2 were significantly higher in total IC/BPS patients than controls. Among IC/BPS patients, IL-8, CXCL10, BDNF, IL-6, and RANTES were significantly higher in patients with HIC than non-HIC patients. * *p* < 0.05, ** *p* < 0.01, *** *p* < 0.001. BDNF, brain-derived neurotrophic factor; CXCL10, C-X-C motif chemokine ligand 10; eotaxin, eotaxin-1; HIC, Hunner’s interstitial cystitis; IC/BPS, interstitial cystitis/bladder pain syndrome; IL-, interleukin-; MCP-1, monocyte chemoattractant protein-1; MIP-1β, macrophage inflammatory protein-1 beta; PGE2, prostaglandin E2; RANTES, regulated upon activation, normally T-expressed, and presumably secreted; TNF-α, tumor necrosis factor-alpha.

The urine biomarkers of patients with IC/BPS (including non-HIC and HIC) and controls are listed in Table 1. Among the 10 examined proteins, the levels of MCP-1, eotaxin, MIP-1β, TNF-α, and PGE2 in IC/BPS patients were significantly different from those recorded in controls. When we compared the urine biomarkers between patients with HIC and non-HIC, IL-8, CXCL10, BDNF, IL-6, and RANTES were significantly higher in HIC than non-HIC patients (Figure 2).

We compared the urine proteins among non-HIC patient subgroups with different MBC (>760 mL or ≤760 mL) and patients with HIC. Patients with HIC had significantly higher urine levels of IL-8, CXCL10, BDNF, eotaxin, IL-6, MIP-1β, and RANTES than non-HIC patients. Interestingly, non-HIC patients with MBC ≤ 760 mL had significantly higher levels of CXCL10, MCP-1, eotaxin, IL-6, MIP-1β, RANTES, and PGE2 than patients with non-HIC with MBC > 760 mL (Table 2).

We also compared the urine biomarkers among non-HIC patient subgroups with different glomerulation grades (≤1 or ≥2) and patients with HIC. Patients with HIC also had significantly higher urine levels of IL-8, CXCL10, BDNF, IL-6, and RANTES than non-HIC patients. Interestingly, only the urine levels of MCP-1 and PGE2 were significantly higher in non-HIC patients with glomerulation grade ≥ 2 than non-HIC patients with GR ≤ 1 (Table 3).

We used ROC curves to analyze each urine biomarker in patients with IC/BPS and controls. The AUC and cut-off values of each biomarker were obtained (Table 4). Based on the cut-off value of each biomarker, the sensitivity, specificity, PPV, and NPV were determined. Among the 10 urine biomarkers, only MIP-1β and TNF-α had an AUC >0.7. In discriminating IC/BPS from controls, IL-8, and TNF-α had a sensitivity > 80%, while CXCL10, MCP-1, eotaxin, MIP-1β, RANTES, TNF-α, and PGE2 had a specificity > 80%. However, only TNF-α had a good sensitivity (99.0%), specificity (92.6%), PPV (98.4%) and NPV (89.3%). In discriminating HIC from non-HIC, we could not find a urine biomarker with a good sensitivity and specificity. Combined, the cut-off values of MIP-1β (0.81) and TNF-α (1.05) can only have a 89.5% sensitivity and 96.4% specificity in identifying IC/BPS from controls, which were even lower than a single TNF-α to predict IC/BPS from controls.

## 4. Discussion

The results of this large cohort study revealed that urine cytokines and chemokines can be useful to discriminate patients with IC/BPS from controls. Among the 10 urine biomarkers examined, MIP-1β and TNF-α had an AUC > 0.7; IL-8 and TNF-α had a sensitivity > 80%; while CXCL10, MCP-1, eotaxin, MIP-1β, RANTES, TNF-α, and PGE2 had a specificity > 80% in identifying IC/BPS patients. However, we could not find a urine biomarker with a good sensitivity and specificity to discriminate HIC from non-HIC. In addition, these urine cytokines and chemokines can also reflect the bladder inflammatory condition, such as small MBC and high glomerulation grade, in patients with IC/BPS.

The chronic pain in IC/BPS may be due to persistent inflammatory reactions in the urinary bladder, which activate afferent sensory nerves and lead to central nervous system sensitization [1,2]. Several pathophysiological mechanisms for the pain of IC/BPS have been proposed; however, none could be proven to result in the clinical characteristics of patients with IC/BPS [9,10,11,12,30,31,32,33]. Recent evidence indicated that IC/BPS is a heterogeneous syndrome, and that ulcer-type and non-ulcer IC/BPS represent different disease entities [34,35]. The diagnosis of IC/BPS may not be reached solely based on clinical and exclusion of other bladder disorders. The development of urine or serum biomarkers used in conjunction with clinical symptoms may increase the rate of early and accurate diagnoses of IC/BPS subtypes [18,23,29].

Currently, most guidelines state that IC/BPS should be classified into HIC and non-HIC. These IC/BPS subtypes are linked to different treatment options and outcome [36]. Among the four non-HIC subgroups, patients with a low glomerulation grade or large MBC exhibited better urodynamic parameters and the highest satisfaction rate, but more medical comorbidities [8]. Thus far, the treatment of non-HIC has not been successful. Use of cystoscopic hydrodistention, intravesical glycosaminoglycan supplementation, intravesical botulinum toxin injection, platelet-rich plasma injection, and, recently, low-energy shock wave therapy has been proposed. Nevertheless, none of these treatment modalities achieved a long-term successful outcome [37].

HIC typically results in a thick, contracted bladder with severely erosive bladder mucosa. Consequently, electrocauterization, laser ablation, partial cystectomy with or without augmentation, or even cystectomy plus ileal conduit diversion have been widely applied. The results of these invasive surgical approaches are usually satisfactory [38]. Our previous study demonstrated that EBV infection was present in 87.5% and 17.4% of bladder specimens obtained from HIC and non-HIC patients, respectively (total IC/BPS: 46.2%). Patients with proven EBV infection had severe bladder pain and restricted bladder capacity [7]. A computed tomography analysis also showed focal or diffused thickness of the bladder wall, suggesting underlying inflammatory cell infiltration, uroepithelial cell denudation, and granulation tissue in the bladder wall [6]. Antiviral therapy may provide therapeutic success without the need for invasive surgery. Therefore, early diagnosis of HIC is mandatory. If urine biomarkers could indicate the presence of HIC prior to performing the cystoscopic procedure, patients with HIC may have a chance for earlier treatment.

In this study, we collected a large number of patients with IC/BPS and their detailed clinical characteristics and bladder conditions after cystoscopic hydrodistention. Therefore, the results of this investigation regarding urine biomarkers are more reliable than those of previous studies. In this study, we found MIP-1β and TNF-α had an AUC >0.7; IL-8 and TNF-α had a sensitivity > 80%; while CXCL10, MCP-1, eotaxin, MIP-1β, RANTES, TNF-α, and PGE2 had a specificity > 80% in identifying IC/BPS patients. These urine cytokines and chemokines play important roles in the diagnosis of IC/BPS and mapping of the clinical characteristics. However, between HIC and non-HIC, although the mean urine biomarkers of IL-8, CXCL10, BDNF, IL-6, and RANTES were significantly higher in HIC than non-HIC patients, a good sensitivity and specificity could not be obtained using urine biomarker level. Among patients with suspected IC/BPS, using urine biomarkers, we could identify suitable patients for further diagnostic tests and to search for HIC. If necessary, therapy should be administered as early as possible to prevent the progression of bladder inflammation.

Previous studies have noted that several urine biomarkers, including CXCL1, CXCL10, CXCL8, MCP-1, IL-6, IL-8, matrix metallopeptidase 8, VEGF, platelet-derived endothelial cell growth factor, nerve growth factor (NGF), BDNF, and TNF-α were increased in patients with HIC [23,25,29,39]. In our previous study, we also found, among cytokines with high diagnostic values (AUC > 0.7), to diagnose ESSIC type 2 IC/BPS from controls, RANTES, MIP-1β, and IL-8 were of higher sensitivity, and MCP-1, CXCL10, and eotaxin were of higher specificity. MCP-1, CXCL10, eotaxin, and RANTES were all positively correlated with the grade of glomerulations and negatively correlated with MBC [23]. The cytokines with high diagnostic values to distinguish IC/BPS and overactive bladder included IL-10, RANTES, eotaxin, CXCL10, IL-12p70, NGF, IL-6, IL-17A, MCP-1, and IL-1RA [30]. However, in this study, only MIP-1β and TNF-α had a high sensitivity and high specificity in identifying IC/BPS from the controls. The discrepancy of results between this study and other studies might be due to different IC/BPS population. Most of the results from previous studies were obtained from small cohorts of patients with IC/BPS, and the clinical diagnosis of IC/BPS was not consistently reached. This study collected a large number of IC/BPS patients, and the clinical characteristics were different among the patients. Therefore, a panel of urine biomarkers could not be reached to identify HIC from total IC/BPS patients.

MCP-1, also termed C-C motif chemokine ligand 2 (CCL2), is a potent chemotactic factor for monocytes. Numerous cell types, including endothelial cells, epithelial cells, fibroblasts, and smooth muscle cells, can produce MCP-1 [40]. It has been reported that MCP-1 is involved in many inflammatory diseases [41,42], (e.g., inflammatory bowel disease, allergic asthma, and rheumatoid arthritis), which are also associated with IC/BPS. In this study, MCP-1 was increased in all patients with IC/BPS, and exhibited the highest levels in patients with small MBC and higher glomerulation grade. This evidence further implies the association of MCP-1 with chronic inflammation in IC/BPS.

Eotaxin, also termed CCL11, acts as a selective chemoattractant of eosinophils [43]. It is implicated in numerous eosinophilic inflammatory diseases [44,45]. Eotaxin is one of the potential urinary biomarkers of IC/BPS [18]. In our previous study, eotaxin demonstrated high specificity for the diagnosis of ESSIC type 2 IC/BPS [23]. In this study, we also found that eotaxin was significantly higher in both HIC and non-HIC with high glomerulation grade and low MBC. The specificity for IC/BPS and HIC were also high. These results suggested that eotaxin-associated inflammation plays an important role in the pathophysiology of HIC and more severe IC/BPS.

MIP-1β is a chemoattractant for natural killer cells, monocytes, and a variety of other immune cells [46]. MIP-1β was elevated in urine collected from patients with overactive bladder (OAB) and was thought to be related to bladder inflammation [47]. However, the exact role of MIP-1β in the pathogenesis of OAB and IC/BPS remains uncertain. In this study, MIP-1β was significantly lower in patients with IC/BPS than controls. However, MIP-1β was significantly higher in patients with HIC than non-HIC patients. It was also significantly associated with a lower MBC in patients with IC/BPS. It is likely that MIP-1β may also be used as a reference biomarker for the diagnosis of HIC.

RANTES is a chemotactic factor for T cells, eosinophils, and basophils; it plays an active role in the recruitment of leukocytes into inflammatory sites [48]. The central role of RANTES in the pathophysiology of neurogenic cystitis has been identified [49]. Anti-RANTES antibodies block mast cell trafficking and subsequently stabilize bladder barrier function. In this study, although the mean urine RANTES levels in patients did not differ from those recorded in controls, increased RANTES urine levels were significantly associated with small MBC and higher glomerulation grade. Significantly higher RANTES urine levels were also noted in patients with HIC versus non-HIC patients. These results indicate that RANTES may be an important target in the pathophysiology and treatment of IC/BPS.

TNF-α is a proinflammatory cytokine that can cause excessive inflammation and bladder damage [50]. The TNF-α levels in the HIC bladder tissue were significantly increased. Mast cell activation with the release of TNF-α elicited an inflammatory response in IC/BPS; therefore, the urine TNF-α levels would be elevated [51]. The results of this study showed that the mean TNF-α urine levels were significantly higher in patients with IC/BPS than controls. With a cut-off value of 1.05, the sensitivity and specificity were very high for the diagnosis of IC/BPS or HIC.

PGE2 has been considered a good candidate biomarker for OAB; nevertheless, its role in IC/BPS remains unclear [20]. In this study, we found that the urine PGE2 levels were significantly higher in patients with IC/BPS than controls, and had a 63.6% sensitivity and an 80% diagnostic specificity for IC/BPS; however, the sensitivity was only 45.8% for HIC. High urine PGE2 levels were significantly associated with a smaller MBC and a higher glomerulation grade. Urine PGE2 alone should not be used as a biomarker for the diagnosis of HIC, but may be considered as a reference biomarker.

Other urine biomarkers, such as IL-6, IL-8, and CXCL10, are increased in patients with IC/BPS, and specifically in patients with HIC [52]. IL-6 is an interleukin that acts as both a proinflammatory cytokine and an anti-inflammatory myokine. IL-8 is a chemoattractant of neutrophils and T cells [53]. It regulates angiogenesis through the direct enhancement of the survival and proliferation of endothelial cells [54]. In addition, IL-8 is essential for normal urothelial growth and survival factors [53]. CXCL10 is a chemotactic factor for monocytes/macrophages and activated T cells [55]. In a human study, urine CXCL10 levels were significantly higher in ulcer-type IC/BPS, but not in non-ulcer IC/BPS, compared with control [39,56]. In the present study, the mean urine levels of IL-6, IL-8, and CXCL10 were not significantly higher than those observed in controls. However, the urine levels of these three chemokines and cytokines were significantly higher in patients with HIC versus non-HIC patients. In addition, urine CXCL10 and IL-6 levels were significantly associated with a lower MBC and higher glomerulation grade in patients with IC/BPS. Based on these results, these three proteins may also be used as biomarkers for the diagnosis of HIC.

The levels of urine cytokines and chemokines were significantly different among the various IC/BPS subgroups. The levels of MCP-1, eotaxin, TNF-α, and PGE2 were significantly higher in patients with IC/BPS than controls. When we divided non-HIC patients into small MBC (≤760 mL) and large MBC (>760 mL), significantly higher urine levels of CXCL10, BDNF, eotaxin, IL-6, MIP-1β, and RANTES were observed in the HIC group versus the other non-HIC subgroups. Meanwhile, we also noted Non-HIC patients with an MBC ≤ 760 mL had seven higher urine biomarkers compared with those with MBC > 760 mL, suggesting a small MBC has higher bladder inflammation in non-HIC patients. Significantly higher urine levels of IL-8, CXCL10, BDNF, IL-6, and RANTES were also noted in patients with HIC compared with non-HIC subgroups divided by high and low glomerulation grades. The reduced MBC and elevated glomerulation grade suggest increased bladder inflammation [15].

Collectively, the urine levels of IL-8, CXCL10, BDNF, eotaxin, IL-6, MIP-1β, and RANTES were significantly increased in patients with HIC than non-HIC patients based on different MBC or glomerulation grades. Most of these cytokines and chemokines are significantly associated with a smaller MBC and higher glomerulation grade. These results suggest that the increased levels of urine cytokines and chemokines are associated with a high grade of bladder inflammation in patients with IC/BPS, and more severe inflammation in patients with HIC. Based on the present findings, in the presence of increased levels of these urine biomarkers, such as CXCL10, BDNF, eotaxin, IL-6, MIP-1β, and RANTES, HIC should be suspected.

There are several limitations of this study. First, the case number is asymmetrical between HIC and non-HIC patients and controls, resulting in a low predictive value. Second, the urine samples were collected and stored over a long period of time, therefore, the urine proteins may have decayed with time and caused a decrease in the urinary concentration. However, the simultaneous investigation of the 10 biomarkers may have reduced the error in protein concentration.

## 5. Conclusions

The results of this study demonstrated that urine cytokines and chemokines may be useful to discriminate patients with IC/BPS from controls. Among the selected 10 urine biomarkers, only MIP-1β and TNF-α had an AUC > 0.7 in discriminating IC/BPS from controls. However, the differences of urine biomarkers in discriminating HIC from non-HIC patients were limited. An elevation in the urine levels of IL-8, CXCL 10, BDNF, IL-6, and RANTES in IC/BPS patients should prompt physicians to consider the diagnosis of HIC, and potentially initiate early treatment.

## Figures and Tables

**Table 1:** The urine biomarkers between IC/BPS subgroup patients and controls.

Urine Biomarker @	IC/BPS (*n* = 309)	Control (*n* = 30)	*p*-Value *	Non-HIC (*n* = 285)	HIC (*n* = 24)	*p*-Value #
IL-8	17.2 ± 25.5	12.5 ± 21.0	0.328	15.9 ± 23.6	34.4 ± 39.7	0.042
CXCL 10	11.7 ± 20.2	13.8 ± 18.4	0.583	10.1 ± 17.4	35.1 ± 38.2	0.011
MCP-1	298 ± 301	147 ± 110	<0.001	299 ± 306	289 ± 239	0.879
BDNF	0.58 ± 0.16	0.55 ± 0.12	0.310	0.57 ± 0.14	0.71 ± 0.30	0.034
Eotaxin	7.65 ± 7.55	4.98 ± 3.7	0.002	7.29 ± 7.05	12.0 ± 11.5	0.064
IL-6	3.48 ± 8.35	1.29 ± 1.35	0.160	2.92 ± 6.96	10.8 ±8.35	0.047
MIP-1β	1.24 ± 1.73	2.52 ±1.82	<0.001	1.18 ± 1.60	1.96 ± 2.80	0.198
RANTES	5.69 ± 8.18	6.04 ± 5.15	0.820	5.30 ± 7.90	10.2 ± 10.1	0.027
TNF-α	1.66 ± 0.38	0.82 ± 0.33	<0.001	1.65 ± 0.35	1.85 ± 0.64	0.145
PGE2	292 ± 241	161 ± 105	<0.001	291 ± 232	302 ± 335	0.882

@: units: pg/mL, * comparison of urine biomarkers between total IC/BPS patients and control, # comparison of urine biomarkers between HIC and non-HIC patients. BDNF, brain-derived neurotrophic factor; CXCL10, C-X-C motif chemokine ligand 10; eotaxin, eotaxin-1; IC/BPS, interstitial cystitis/bladder pain syndrome; IL-, interleukin-; MCP-1, monocyte chemoattractant protein-1; MIP-1β, macrophage inflammatory protein-1 beta; PGE2, prostaglandin E2; RANTES, regulated upon activation, normally T-expressed, and presumably secreted; TNF-α, tumor necrosis factor-alpha.

**Table 2:** The urine biomarkers among IC/BPS subgroup patients with different maximal bladder capacity and Hunner’s IC patients.

Urine Biomarkers @	(A) IC/BPS MBC > 760 mL (*n* = 125)	(B) IC/BPS MBC ≤ 760 mL (*n* = 160)	(C) Ulcer IC/BPS (*n* = 24)	*p*-Value A vs. B vs. C	*p*-Value A vs. B
IL-8	15.2 ± 25.7	16.5 ± 21.9	34.4 ± 39.7 *	0.043	0.634
CXCL 10	6.45 ± 11.9	13.0 ± 20.3	35.1 ± 38.2 *	0.001	0.001
MCP-1	227 ± 222 *	356 ± 349 *	289 ± 239	<0.001	<0.001
BDNF	0.58 ± 0.13	0.56 ± 0.14	0.71 ± 0.3 *	0.020	0.246
Eotaxin	5.93 ± 5.9	8.35 ± 7.69 *	12.0 ± 11.5	0.010	0.003
IL-6	2.0 ± 6.06	3.64 ± 7.53 *	10.8 ± 17.4	0.022	0.043
MIP-1β	0.86 ± 1.23 *	1.42 ± 1.81 *	1.96 ± 2.80	0.037	0.002
RANTES	4.09 ± 8.4	6.26 ± 7.36	10.2 ± 10.1 *	0.007	0.023
TNF-α	1.64 ± 0.35 *	1.65 ± 0.35 *	1.85 ± 0.64 *	0.182	0.651
PGE2	253 ± 213 *	322 ± 242 *	302 ± 335 *	0.138	0.014

@: units: pg/mL, * *p* < 0.05 comparison between IC/BPS subgroup and control; MBC, maximal bladder capacity; other abbreviations: same as Table 1.

**Table 3:** The urine biomarkers among IC/BPS subgroup patients with different grade of glomerulations or Hunner’s lesion.

Urine Biomarkers @	(A) GR ≤ 1 (*n* = 155)	(B) GR > 1 (*n* = 130)	(C) Hunner’s IC (*n* = 24)	*p*-Value A vs. B vs. C	*p*-Value A vs. B
IL-8	17.7 ± 27.2	13.7 ± 18.5	34.4 ± 39.7 *	0.026	0.158
CXCL 10	8.7 ± 16.1	11.8 ± 18.7	35.1 ± 38.2 *	0.004	0.141
MCP-1	238 ± 227 *	370 ± 366 *	289 ± 239	0.001	<0.001
BDNF	0.57 ± 0.14	0.56 ± 0.14	0.71 ± 0.3 *	0.023	0.518
Eotaxin	6.87 ± 6.78	7.8 ± 7.36 *	12.0 ± 11.5	0.058	0.270
IL-6	2.39 ± 5.71	3.55 ± 8.2 *	10.8 ± 17.4	0.028	0.162
MIP-1β	1.15 ± 1.67 *	1.22 ± 1.53 *	1.96 ± 2.80	0.272	0.715
RANTES	4.63 ± 8.33	6.11 ± 7.3	10.2 ± 10.1 *	0.017	0.115
TNF-α	1.64 ± 0.32 *	1.65 ± 0.38 *	1.85 ± 0.64 *	0.189	0.780
PGE2	266 ± 226 *	323 ± 236 *	302 ± 335 *	0.144	0.042

* Comparison between IC/BPS subgroup and controls, GR: grade of glomerulation. @: units: pg/mL; other abbreviations: same as Table 1.

**Table 4:** The receiver operating characteristics of urine biomarkers in diagnosis of IC/BPS patients and Hunner’s IC patients.

Urine Cytokine @	AUC	Cut-Off Value	Total IC Sensitivity	Total IC Specificity	Total IC PPV	Total IC NPV	HIC Sensitivity *	HIC Specificity *	HIC PPV	HIC NPV
IL-8	0.587	2.100	80.6%	40.0%	93.3%	16.7%	95.8%	20.7%	9.2%	98.3%
CXCL 10	0.590	1.595	32.7%	90.0%	97.1%	11.5%	12.5%	65.6%	3.0%	89.9%
MCP-1	0.639	283.1	35.9%	93.3%	98.2%	12.4%	41.7%	64.6%	9.0%	92.9%
BDNF	0.551	0.543	57.3%	66.7%	94.7%	13.2%	87.5%	45.3%	11.9%	97.7%
Eotaxin	0.587	12.50	21.0%	96.7%	98.5%	10.6%	41.7%	80.7%	15.4%	94.3%
IL-6	0.534	0.515	38.2%	83.3%	95.9%	11.6%	16.7%	60.0%	3.4%	89.5%
MIP-1β	0.774	0.810	60.5%	100%	100%	19.7%	50.0%	38.6%	6.4%	90.2%
RANTES	0.636	1.495	36.9%	100%	100%	13.3%	25.0%	62.1%	5.3%	90.8%
TNF-α	0.920	1.050	99.0%	92.6%	98.4%	89.3%	100%	1.1%	7.8%	100%
PGE2	0.679	175.4	63.6%	80.0%	97.0%	17.6%	45.8%	34.9%	5.6%	88.4%

@: units: pg/mL; * HIC sensitivity and specificity are calculated HIC from total IC/BPS patients; AUC, area under curve; Total IC, including non-HIC and HIC; HIC, Hunner’s IC; PPV, positive predictive value; NPV, negative predictive value, other abbreviations: same as Table 1.

## Data Availability

Data are available upon request from the corresponding authors.

## Abbreviations

IC/BPS	interstitial cystitis/bladder pain syndrome
HIC	Hunner’s ulcer interstitial cystitis
MBC	maximal bladder capacity under anesthesia
IL-8	interleukin 8
CXCL10	C-X-C motif chemokine ligand 10
MCP-1	monocyte chemoattractant protein 1
NGF	nerve growth factor
BDNF	brain-derived neurotrophic factor
IL-2	interleukin 2
IL-6	interleukin 6
MIP-1β	macrophage inflammatory protein-1β
RANTES	regulated upon activation, normally T-expressed, and presumably secreted
TNF-α	tumor necrosis factor-alpha
PGE2	prostaglandin E2
ICSI	interstitial cystitis symptom index
ICPI	interstitial cystitis problem index
VAS	visual analog scale
PPV	positive predictive value
NPV	negative predictive value
